# Emergence of *BRCA* Reversion Mutations in Prostate Cancer Prior to PARP Inhibitor Exposure: Clinical and Therapeutic Implications

**DOI:** 10.1002/cam4.71489

**Published:** 2026-01-15

**Authors:** Douglas I. Lin, Elizabeth Lawrence, Natalie Danziger, Huihui Ye, Brennan Decker, Ole Gjoerup, Ryon P. Graf, Jeffrey S. Ross, Richard S. P. Huang, Julia A. Elvin, Douglas A. Mata, Rana R. McKay

**Affiliations:** ^1^ Foundation Medicine, Inc. Boston Massachusetts USA; ^2^ Cedars‐Sinai Medical Center Los Angeles California USA; ^3^ Upstate University Medical Center Syracuse New York USA; ^4^ University of California‐San Diego San Diego California USA

**Keywords:** BRCA1/2, liquid biopsy, PARP inhibitor, prostate cancer, reversion

## Abstract

**Background:**

Inactivating *BRCA1/2* mutations confer sensitivity to poly(ADP‐ribose) polymerase inhibitors (PARPi) in prostate cancer (PCA). However, secondary *BRCA1/2* reversion mutations (*BRCA*rev) can restore *BRCA* function and mediate acquired PARPi resistance. While *BRCA*rev typically arise under PARPi selective pressure, their occurrence in PARPi‐naïve settings remains incompletely understood. We sought to characterize the frequency, clinical context, and therapeutic correlates of *BRCA*rev detection in men with advanced PCA, including those without prior PARPi exposure.

**Methods:**

We restrospectively analyzed clinical liquid biopsy results from men with PCA using FoundationOneLiquid CDx between January and December 2023. *BRCA*rev were defined as sequence alterations predicted to restore open reading frame of *BRCA1* or *BRCA2* harboring pathogenic inactivating variants. Clinical and treatment histories, including chemotherapy and PARPi exposure, were abstracted from medical records.

**Results:**

Over a one‐year period, we identified 10 PCA patients with inactivating *BRCA1/2* alterations, *BRCA*rev and available treatment history. *BRCA*rev were detected in three of 10 (30%) patients who had not received prior PARPi therapy. All three PARP‐naïve patients had previously received chemotherapy (platinum or docetaxel) and radiation, and each exhibited multiple *BRCA*rev events. The remaining 7 patients (70%) had prior olaparib exposure. Among these, the duration of PARPi response was longer in chemo‐naïve patients compared with previously treated with chemotherapy (median: 22 vs. 7.5 months, respectively).

**Conclusions:**

*BRCA*rev can emerge in PCA in the absence of prior PARPi therapy, particularly following exposure to cytotoxic chemotherapy and radiation. These findings support that DNA‐damaging therapies may promote *BRCA*rev formation, potentially predisposing to primary PARPi resistance. Early integration of PARPi therapy before chemotherapy may enhance clinical benefit and circumvent emergence of primary resistance.

## 1 Introduction

Pathogenic alterations in *BRCA1/2 (*collectively referred as *BRCA*) occur in up 5%–10% of prostate cancers (PCA), particularly in the metastatic castration‐resistance setting (mCRPC) [1, 2, 3, 4]. *BRCA* loss of function results in homologous recombination deficiency (HRD), which impairs DNA double‐strand break repair and renders tumor cells sensitive to poly(ADP‐ribose) polymerase inhibitors (PARPi) through a synthetic lethality mechanism [5, 6]. Based on multiple Phase III clinical trials, including PROfound, TRITON3, and TALAPRO‐2, the U.S. Food and Drug Administration (FDA) has approved PARPi agents, such as olaparib, rucaparib, niraparib and talazoparib, for the treatment of *BRCA*‐mutated, metastatic castration‐resistant PCA [7, 8, 9, 10, 11].

Despite an initial clinical benefit, resistance to PARPi almost inevitably develops. One of the most characterized resistance mechanisms involves *BRCA1/2* reversion mutations (*BRCA*rev), defined as secondary somatic alterations that restore the open reading frame or functional protein expression of a previously inactivating *BRCA* mutation [12, 13, 14]. These reversions restore homologous recombination repair proficiency and eliminate susceptibility to PARPi [12]. *BRCA*rev most commonly arise as short insertions, deletions, or point mutations that correct the original inactivating defect, though larger rearrangements have also been described [15, 16].

In this context, primary PARPi resistance refers to the lack of initial clinical response or rapid progression despite adequate therapy exposure, whereas acquired (secondary) resistance denotes disease progression after an initial response [17]. While most *BRCA*rev events are associated with acquired PARPi resistance under therapeutic selective pressure, emerging evidence indicates that DNA‐damaging agents such as platinum‐based chemotherapy or radiation may also induce *BRCA*rev in the absence of prior PARPi exposure, potentially leading to primary PARPi resistance [14]. Notably, PCAs with homozygous, biallelic *BRCA* loss (complete gene deletion) [18] or HRD arising through irreversible mechanisms (e.g., epigenetic silencing due to promoter methylation or inactivating complex structural rearrangements) are less likely to acquire reversion mutations and may exhibit a more durable PARPi response [16, 18].

Although *BRCA*rev have been extensively reported in ovarian, breast and pancreatic cancers [13, 14, 19], their clinical context and temporal evolution in PCA remain poorly understood. Prospective analyses from the TRITON2 study revealed the emergence of *BRCA*rev in up to 40% of mCRPC patients at the time of PARPi progression [20], but systematic evaluation of BRCArev in PARP‐naïve PCAs is lacking. Moreover, the impact of prior cytotoxic therapy on the development of *BRCA*rev and subsequent PARPi treatment duration remains unclear [20].

We hypothesized that *BRCA*rev may emerge in advanced PCA prior to PARPi exposure, particularly following DNA‐damaging chemotherapy or radiotherapy, and that such events could confer primary resistance to PARPi. The objectives of this study were therefore to (i) determine the prevalence of *BRCA*rev in a real‐world cohort of men with advanced *BRCA*‐mutated PCA, including in PARPi‐naïve patients; (ii) characterize the clinical and therapeutic contexts associated with *BRCA*rev detection; and (iii) explore the relationship between prior cytotoxic chemotherapy and subsequent PARPi response duration. By integrating genomic and clinical data, this study aims to clarify how treatment chronology influences the emergence of *BRCA*rev and the effectiveness of PARPi in advanced PCA.

## 2 Methods

### 2.1 Study Design and Cohort Selection

This retrospective, observational study included adult male patients (> 18 years) with a confirmed diagnosis of PCA. Approval of this study, including a waiver of informed consent and Health Insurance Portability and Accountability Act waiver of authorization, was obtained from the Western Institutional Review Board (Protocol No. 20152817). This study was performed in accordance with the principles of the Declaration of Helsinki. Men with advanced or metastatic PCA patients who underwent liquid‐based comprehensive genomic profiling (CGP) between January 1, 2023 and December 31, 2023 during clinical care across various institutions across North America using FoundationOneLiquid CDx (F1LCDx, Foundation Medicine Inc. Boston, MA) were retrospectively evaluated.

Liquid biopsy results were screened for the presence of pathogenic, inactivating *BRCA1/2* alterations and corresponding secondary *BRCA*rev alterations. Clinical and treatment histories were extracted from medical records and/or from treating oncologists' notes accompanying test requisitions. Cases without accompanying treatment history were excluded from the final *BRCA*rev patient cohort.

Of 25 PCA patients in whom *BRCA*rev identified during the study period, 15 were excluded due the lack of treatment histories, resulting in a final analytic cohort of 10 *BRCA*rev patients with documented clinicopathologic data. Randomization and blinding were not applicable due to the retrospective observational design. Response or progression on PARPi was determined based on prostate‐specific antigen (PSA) dynamics (≥ 50% reduction or increase from baseline) and/or radiographic evidence of response or progression per treating physician assessment.

### 2.2 Liquid Biopsy Collection, Processing and Sequencing

Peripheral whole blood (2 × 8.5 mL) was collected into Streck Cell‐Free DNA BCT tubes as part of routine clinical care. Samples were shipped at ambient temperature and processed at Foundation Medicine's CLIA‐certified, CAP‐accredited, and New York State‐approved laboratory. Plasma was isolated via centrifugation and used for the extraction of circulating cell‐free DNA (cfDNA).

Library preparation and hybrid capture‐based next‐generation sequencing (NGS) were performed as previously described [21]. Briefly, cfDNA underwent end‐repair, A‐tailing, and ligation to barcoded sequencing adapters containing unique molecular identifiers (UMIs). Hybrid capture was conducted using a custom‐designed probe set targeting exonic regions of 324 cancer‐related genes, including *BRCA1* and *BRCA2*, as well as select intronic regions frequently involved in genomic rearrangements. Captured libraries were sequenced on the Illumina NovaSeq platform to high uniform depth (median unique coverage > 5000×).

Sequencing data were analyzed using Foundation Medicine's proprietary bioinformatics pipeline to identify base substitutions, short insertions/deletions (indels), copy number alterations, and genomic rearrangements. Variant calling incorporated local realignment, read deduplication, and error suppression using UMIs to enhance sensitivity and specificity for low frequency variants. Tumor fraction (TF) was estimated using a combination of genome‐wide copy number modeling and variant allele frequency (VAF) distribution.

### 2.3 Identification of Pathogenic *BRCA1/2* Alterations and Reversion Mutations

Pathogenic *BRCA1* and *BRCA2* alterations and corresponding *BRCA*rev were detected using F1LCDx. Somatic versus germline origin predictions for pathogenic *BRCA1/2* mutations were determined using a proprietary algorithm that integrates VAF, copy number modeling, and circulating tumor DNA tumor fraction (ctDNA TF). An alteration was classified as a *BRCA*rev mutation if it was predicted to restore BRCA1/2 function by nullifying the effect of a pathogenic *BRCA1/2* alteration. *BRCA*rev detection is contingent upon the presence of a pathogenic *BRCA1/2* alteration in the sample.

Initial *BRCA*rev identification was performed using a computational algorithm that evaluates the presence of two *BRCA* variants and tests for six distinct reversion mechanisms: (1) pathogenic variant encompassed by an in‐frame deletion; (2) pathogenic variant replaced by a missense substitution; (3) frameshift pathogenic variant corrected by frame‐restoring indel; (4) exon containing a pathogenic variant predicted to undergo skipping due to splice site mutation; (5) pathogenic variant removed by an exon‐level deletion; and (6) biallelic deletion of the region containing the pathogenic variant. Following computational flagging, all candidate *BRCA*rev events underwent manual review by scientists and molecular pathologists to confirm the biological plausibility and ensure final classification accuracy.

To exclude that *BRCA*rev were derived from clonal hematopoiesis (CH), we applied the investigational short variant origin prediction (VOP) algorithm developed for the F1LCDx assay. This machine learning model incorporates fragmentomics and other sequencing features and was trained on liquid biopsy samples with matched‐depth sequencing of plasma and white blood cells. The algorithm outputs probabilistic origin classifications (germline, tumor‐somatic, or CH). A high‐confidence threshold (probability > 0.95) was used to designate CH origin and exclude such variants from the *BRCA*rev cohort.

## 3 Results

### 3.1 Overall *BRCA*rev Prostate Cancer Cohort

Between January and December 2023, a total of 416 men with PCA harbored a pathogenic *BRCA1/2* alteration after undergoing blood‐based CGP via liquid biopsy (F1LCDx). Among these, 25 patients (6%) co‐harbored *BRCA1/2* variants predicted to restore gene function (*BRCA*rev). Of these, 10 patients (40%) patients had clinical and treatment histories available and were included in the final *BRCA*rev analysis cohort.

Pathogenic alterations involved *BRCA2* in 9 of 10 patients (90%) and *BRCA1* in one patient (10%). Multiple *BRCA*rev mutations were observed per sample in 9 of 10 patients (90%) (Table 1). All *BRCA*rev events were classified as somatic and tumor‐derived, with no evidence of CH origin based on an investigational short variant VOP algorithm integrated into the F1LCDx pipeline.

**TABLE 1:** Clinicopathologic features of advanced patients with PCA and *BRCA1/2* reversion mutations (*n* = 10) in the absence (*n* = 3, PARPi‐naïve, patients #1–3) or presence of prior PARPi therapy (*n* = 7, all olaparib‐treated, patients #4–10).

n	Original *BRCA1/2* mutation	*BRCA1/2* reversion mutations	PCA type	Prior PARPi	Duration of PARPi	Initial PARPi response	Castration resistant^a^	Prior chemo	Prior XRT	Progression after PARPi
1	*BRCA2* T2542fs*9	T2542K, K2538fs*14, A2534fs*18	NE	None	N/A	N/A	No	Yes, platinum	Yes	N/A
2	BRCA1 K459fs*15	K459_V477 > NLGKPIGRRQASP, K459_K467 > NLGIVFP, E404_H476del, A407_Y465del, K459_Y465 > NLGN, K459_K501 > NLGKPIGRRQASP, D435_K468 > E, E445_S551del, K459_V477 > NLGKPIGRRQASPI, G394_D522 > Y, K459_K468 > NL, K459_Y465 > NLGKP, K459_K467 > NLG, S454_G462del, K459_L474 > NLGKPIGR, D458_I460del, S454_I460 > RQ, H448_Y465del, N455_S470del, K459_A469 > NLG, K459_A469 > NLGKPIGR, K459_P472 > NLGKPIGRRQASK, K459_R466 > NLG, K459_Y465 > *N*, K459_S470 > NLGKPIG, E457_F461del, D458_G462del, E418_F461del, V447_R466del, S451_I460del, E453_G484del, D414_R466del, N319_Y465del, K459_L474 > NLGKPIGRRQASP, K459_L471 > NLGKPIGRRQAS, K459_N480 > NLGKLK, K459_L481 > NLGKPIGRRQASP	ACA	None	N/A	N/A	Yes	Yes, docetaxel + platinum	Yes	N/A
3	BRCA2 L2092fs*7	F1921_V2171del, F2000_Q2164del, E2082_L2092 > LSIV	ACA	None	N/A	N/A	Yes	Yes, docetaxel	Yes	N/A
4	*BRCA2* K2547fs*4	Y2543_I2550 > Q, K2547_K2551 > *N*, C2549fs*4	ACA	Olaparib	24 months	Yes	Yes	No	No	No
5	*BRCA2* E2565fs*80	H2563_F2568 > LHC, E2565_G2569 > VV, E2565_T2575 > VVR	ACA	Olaparib	10 months	Yes	Yes	No	No	Yes
6	*BRCA2* F2560fs*5	F2560L, Q2561fs*87, H2563fs*5	ACA	Olaparib	9 months	Yes	Yes	Yes, docetaxel	No	Yes
7	*BRCA2* Y1710fs*1	I1418_S1770 > T, I1633_S1741del, C1675_S1848del, A1708_S1720del, D1709_A1725del	ACA	Olaparib	6 months	No	Yes	Yes, docetaxel	No	Yes
8	*BRCA2* E1035*	K985_E1036del, E1035L, E1035S	ACA	Olaparib	5 months	No	Yes	Yes, docetaxel	No	Yes
9	*BRCA2* Q2157fs*18	V2151_V2171del, S2156_Q2157delinsL	ACA	Olaparib	13 months	Yes	Yes	Yes, docetaxel	No	Yes
10	*BRCA2* P999fs*44	P999_K1018 > QFQITVLEVASEQ	ACA	Olaparib	22 months	Yes	Yes	No	Yes	Yes

Abbreviations: ACA, adenocarcinoma; N/A, not applicable; NE, neuroendocrine; PCA, prostate cancer; XRT, radiation.

^a^ Castration resistant at time of *BRCA*rev detection.

**FIGURE 1:**
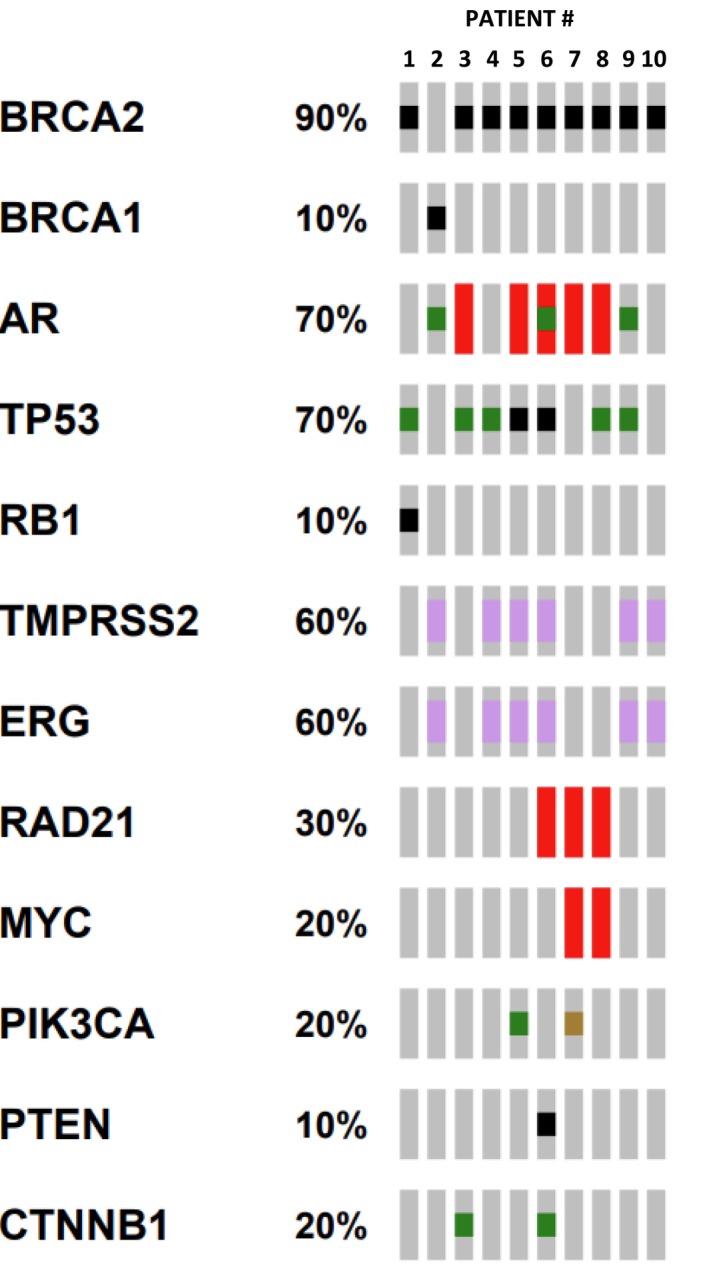
Oncoprint demonstrating co‐occurring alterations within liquid biopsies of 10 *BRCA*‐mutated patients with *BRCA*rev. Each column represents one patient. Patients #1–3 are PARPi‐naïve. Patients #4–10 are PARPi‐treated. Black squares = truncating mutation; green squares = missense mutation; marron square = short variant inframe insertion; red rectangles = amplification; purple rectangles = fusion.

Common co‐occurring alterations included *AR* (70%), *TP53* (70%), *TMPRSS2‐ERG* fusion (60%) (Figure 1). None of the patients exhibited MSI‐H status or pathogenic alterations in *POLE, SPOP*, DNA mismatch repair genes (*MSH2, MSH6, PMS2, MLH1*) (Figure 1).

*BRCA*rev mutations were detected in 3 of 10 patients who had not received prior PARP inhibitor (PARPi) therapy (PARPi‐naïve group) and in 7 of 10 patients (70%) with prior PARPi exposure (all olaparib) (Table 1). Subsequent analyses were stratified by treatment exposure history.

### 3.2 PARPi‐Naïve Patients With *BRCA* Reversion Mutations

Three patients (Table 1, Patients #1–3) exhibited somatic *BRCA*rev in the absence of prior PARPi therapy. All three had previously received cytotoxic chemotherapy (platinum or docetaxel) and radiation before genomic testing. Radiation was directed to bone metastases in all three cases, and to primary tumor sites in two patients.

One patient (Patient 1) presented with de novo high‐stage small cell neuroendocrine carcinoma of the prostate without prior androgen deprivation therapy (Table 1). This diagnosis was supported by concurrent *TP53* and *RB1* alterations and absence of *AR* pathway resistance mutations (Figure 1). The other two patients (Patients 2 and 3, Table 1) had mCRPC, which was supported by the presence of *AR* resistance‐associated alterations (Figure 1).

Two of the three patients harbored pathogenic *BRCA2* truncating variants (Table 1), T2542fs*9 (Patient 1; VAF 45%) and L2092fs*7 (Patient 3; VAF 52%), which were of predicted germline origin. The third patient (Patient 2) harbored a pathogenic *BRCA1* K459fs*15 mutation (VAF 22%) of unknown germline status.

**FIGURE 2:**
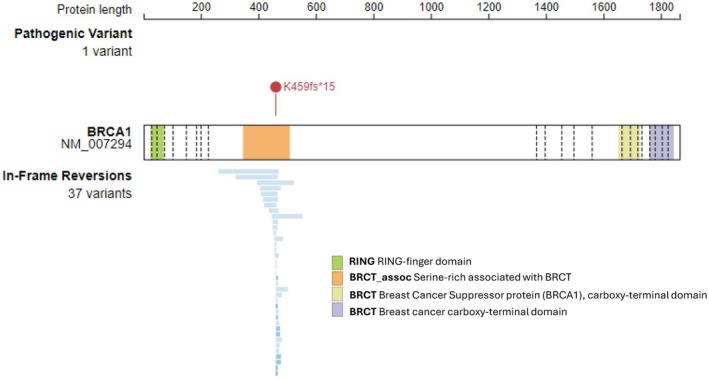
Schematic of the primary pathogenic *BRCA1* K459fs*15 mutation and associated *BRCA1* reversion mutations (*n* = 37) for one PARPi‐naïve prostate cancer patient (Patient 2). Blue bars denote amino acid span of *BRCA1* reversion mutations.

**FIGURE 3:**
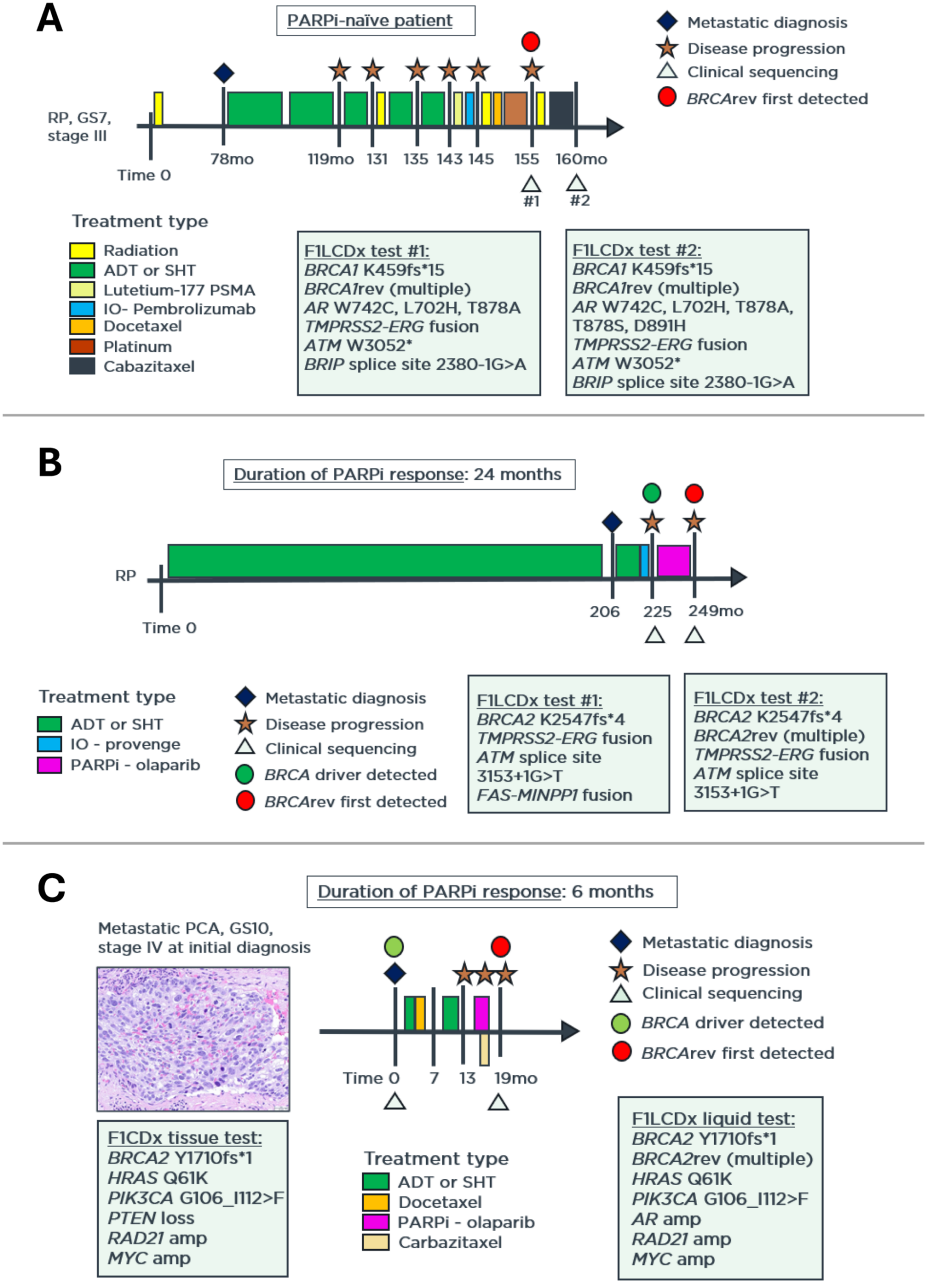
Treatment timelines for patients with PCA and *BRCA*rev. (A) PARPi‐naïve patient (Patient 2). (B) PARPi‐treated patient with no prior chemotherapy or radiation (Patient 4). (C) PARPi‐treated patient with prior chemotherapy (Patient 7). ADT, androgen deprivation therapy; GS, Gleason score; IO, immunotherapy; RP, radical prostatectomy; SHT, secondary hormone therapy.

Multiple *BRCA* reversion mutations were identified per patient (*n* = 3, *n* = 37, and *n* = 3, respectively), all detected after chemotherapy and/or radiation exposure. The cumulative variant allele frequencies (VAF) of *BRCA*rev in these patients were 1.6%, 9.5% and 8%, respectively, consistent with subclonal or polyclonal emergence of reversion events. A representative schematic of the primary pathogenic *BRCA1* alteration and associated *BRCA*rev variants from a PARPi‐naïve patient (Patient 2) is shown in Figure 2, and the patient's treatment timeline in Figure 3A.

### 3.3 Patients With *BRCA* Reversion Mutations and Prior PARPi Exposure

Seven patients (Patients 4–10, Table 1) developed *BRCA*rev following treatment with olaparib (300 mg, twice a day, by mouth). All seven harbored inactivating *BRCA2* mutations as the primary pathogenic event: K2547fs*4 (Patient 4; 2.3% VAF), E2565fs*80 (Patient 5; 21.1% VAF), F2560fs*5 (Patient 6; 38% VAF), Y1710fs*1 (Patient 7; 87.5% VAF), E1035* (Patient #8; 49.8% VAF), Q2157fs*18 (Patient 9; 50% VAF), and P999fs*44 (Patient 10; 58.9% VAF). The latter four of these variants were predicted to be germline in origin. One patient (Patient 10) exhibited a single *BRCA*rev mutation, while the remaining six patients displayed multiple independent reversion mutations (*n* = 3, 3, 3, 5, 3, 2 per case, respectively). The cumulative VAF percentage of *BRCArev* accross the seven patients ranged from 0.2% to 36.2% (median 14.3%), suggesting variable degrees of subclonal *BRCA* reversions within the ctDNA compartment.

All seven PARPi‐treated patients had mCRPC at the time of *BRCA*rev detection (Table 1). Four of the seven (57%) had been previously pre‐treated with chemotherapy (all docetaxel) prior to PARPi initiation, and two had undergone prior radiation therapy (Table 1). The duration of PARPi treatment before detection of *BRCA*rev ranged from 5 to 24 months.

**FIGURE 4:**
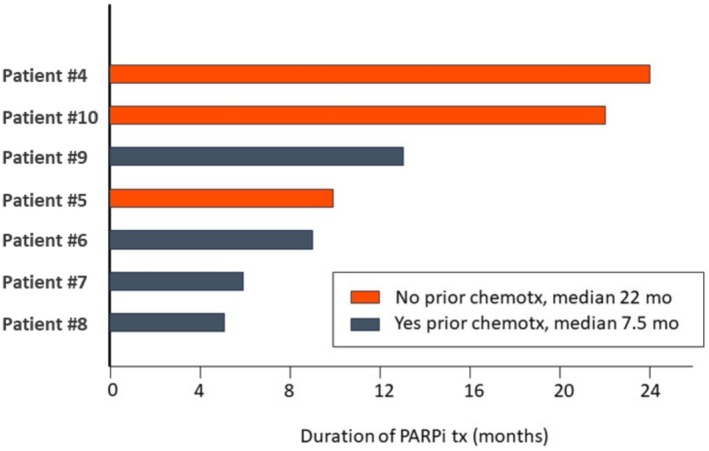
Swimmer plot of 7 *BRCA*‐mutated patients who developed *BRCA*rev following PARPi (all olaparib) therapy. Duration of PARPi treatment was compared between patients that were pre‐treated with chemotherapy (all docetaxel) versus patients that received no prior chemotherapy before PARPi.

Notably, the duration of PARPi response was shorter in patients with prior chemotherapy exposure compared with those who were chemo‐naïve (median 7.5 vs. 22 months, respectively Figure 4). Representative clinical timelines and molecular profiles for two illustrative PARPi‐treated patients, one chemotherapy‐naïve (Patient 4) and one chemotherapy‐pretreated (Patient 7) are shown in Figure 3B,C.

## 4 Discussion

Current guidelines recommend germline and/or somatic testing for homologous recombination gene mutations, including *BRCA1* and *BRCA2*, in all patients with metastatic PCA to identify candidates for PARPi therapy [22]. In this study, we identified a subset of patients with *BRCA*‐mutated PCA who developed *BRCA* reversion mutations in the absence of PARPi therapy, suggesting that exposure to chemotherapy and/or radiation may induce or select for these reversions. These findings have potential implications for treatment sequencing and resistance mechanisms in advanced PCA.

### 4.1 Chemotherapy and Radiation as Potential Drivers of *BRCA* Reversion

Our analysis demonstrated that *BRCA* reversions were somatic and tumor‐derived, often emerging after exposure to platinum‐based or taxane chemotherapy and radiation. These reversions are predicted to abrogate the effect of the original, pathogenic *BRCA* *1/2* mutations and restore the open reading frame and BRCA1/2 protein function, consistent with mechanistic basis or PARPi resistance reported in ovarian, breast and PCAs [12]. Prior studies have established that *BRCA*rev can restore homologous recombination proficiency and confer cross‐resistance to platinum and PARPi therapies [23, 24].

Notably, in the TRITON2 clinical trial, no baseline *BRCA*rev was detected in any patient prior to PARPi treatment [20], likely because enrolled patients were platinum‐naïve. In contrast, our study observed baseline *BRCA*rev in three PARPi‐naïve patients, all of whom had received chemotherapy and radiation [20]. This suggests that cytotoxic DNA‐damaging therapies may create selective pressure for *BRCA* reversion clones before PARPi exposure. Prior, rare case reports describing *BRCA*rev emergence after platinum exposure but before PARPi therapy support this hypothesis [19, 25].

### 4.2 Implications for Treatment Sequencing and Clinical Practice

Our findings support the concept that earlier introduction of PARPi therapy, before chemotherapy, may preserve therapeutic sensitivity in patients with *BRCA1/2*‐mutated PCA. In our cohort, patients who were chemotherapy‐naïve prior to PARPi exhibited longer PARPi response durations than those pretreated with chemotherapy (median 22 vs. 7.5 months). This observation aligns with emerging evidence from the TRITON‐3 trial, in which a subset analysis demonstrated efficacy of rucaparib in *BRCA*‐mutated mCRPC before docetaxel chemotherapy [10]. Similarly, olaparib has shown durable activity in pre‐docetaxel mCRPC [11], and recent approvals of niraparib plus abiraterone and talazoparib plus enzalutamide provide additional options for earlier HRR‐targeted treatment [7, 8].

Collectively, these data suggest that PARPi use prior to cytotoxic chemotherapy may delay or prevent the emergence of *BRCA*rev clones and improve therapeutic outcomes. Conversely, in heavily pre‐treated, PARPi‐naïve patients, screening for *BRCA*rev before PARPi initiation could identify individuals at risk for primary PARPi resistance and guide alternative treatment strategies [25, 26].

### 4.3 Clinical and Translational Relevance of Liquid Biopsy

Liquid biopsy via peripheral blood offers a minimally invasive approach to detect *BRCA*rev mutations and monitor clonal evolution in real time. This is particularly relevant in PCA, where bone‐predominant metastases often preclude repeat tissue sampling for molecular testing [27]. Circulating tumor DNA (ctDNA) assays, such as F1LCDx can capture temporal and spatial heterogeneity across metastatic sites, enabling clinicians to identify *BRCA* reversion mutations that emerge under therapeutic pressure [28]. As shown in our cohort, *BRCA*rev often arise late in the disease course, potentially after multiple lines of therapy, highlighting the limitations of relying on archival tissue for molecular decision making.

Integrating ctDNA profiling into standard care could therefore improve treatment stratification, resistance monitoring, and clinical trial enrollment. For example, identifying *BRCA* reversions pre‐PARPi could redirect patients towards alternative therapies or combination trials targeting secondary resistance mechanisms.

### 4.4 Limitations and Future Directions

This study is limited by its retrospective design and small sample size, which constrain definitive statistical conclusions. Sequetial pre‐ and post‐treatment samples were not available to confirm the precise timing of *BRCA*rev emergence. Nonetheless, the temporal association between chemotherapy/radiation and reversion detection, together with prior case‐based evidence, supports the plausibility of therapy‐induced reversion as a mechanism of PARPi resistance. Future prospective, longitudinal studies incorporating serial ctDNA testing before and after treatment are warranted to confirm these findings.

While novel therapeutic combinations (e.g., ATR, WEE1, USP1, or POLθ inhibitors) are under investigation to overcome PARPi resistance [29, 30, 31, 32, 33], such approaches remain experimental and were beyond the scope of our study. Their relevance may become more pronounced once *BRCA*rev can be readily identified and longitudinally monitored in clinical practice.

### 4.5 Conclusions

In summary, our study provides evidence that *BRCA* reversions can arise in *BRCA1/2*‐mutated PCA in the absence of prior PARP inhibitor therapy, likely as an adaptive response to chemotherapy and/or radiation. These findings suggest that prior cytotoxic exposure may promote primary PARPi resistance and shorten subsequent treatment benefit. Early use of PARPi, before chemotherapy, may therefore optimize outcomes for patients with *BRCA*‐altered metastatic PCA.

Routine incorporation of liquid biopsy‐based *BRCA*rev testing before and during therapy could refine patient selection, guide sequencing strategies, and enable real‐time monitoring of tumor evolution. Finally, enhanced clinician education and improved molecular report annotation, explicitly distinguishing *BRCA*‐sensitizing from *BRCA* resistance variants, are needed to ensure accurate interpretation and optimal use of precision oncology data in clinical care.

## Author Contributions

**Douglas I. Lin:** conceptualization (equal), data curation (equal), formal analysis (equal), investigation (equal), project administration (equal), supervision (equal), visualization (equal), writing – original draft (equal), writing – review and editing (equal). **Elizabeth Lawrence:** data curation (equal), investigation (equal), project administration (equal), visualization (equal), writing – review and editing (equal). **Natalie Danziger:** data curation (equal), investigation (equal), visualization (equal), writing – review and editing (equal). **Huihui Ye:** investigation (equal), writing – review and editing (equal). **Brennan Decker:** investigation (equal), validation (equal), visualization (equal), writing – review and editing (equal). **Ole Gjoerup:** investigation (equal), resources (equal), writing – review and editing (equal). **Ryon P. Graf:** investigation (equal), writing – review and editing (equal). **Jeffrey S. Ross:** investigation (equal), supervision (equal), writing – review and editing (equal). **Richard S. P. Huang:** investigation (equal), writing – original draft (equal). **Julia A. Elvin:** investigation (equal), supervision (equal), writing – review and editing (equal). **Douglas A. Mata:** investigation (equal), supervision (equal), writing – review and editing (equal). **Rana R. McKay:** investigation (equal), writing – review and editing (equal).

## Funding

The authors have nothing to report.

## Ethics Statement

Approval for this study, including a waiver of informed consent and a Health Insurance Portability and Accountability Act waiver of authorization, was obtained from the Western Institutional Review Board, Protocol No. 20152817. The study was performed in accordance with the Declaration of Helsinki.

## Conflicts of Interest

H.Y. and R.M. declare no conflicts of interest. At the time of the study, all remaining authors were full‐time employees of Foundation Medicine Inc. a whole subsidiary of Roche.

## Data Availability

All relevant data were provided within the article. If needed, additional details may also be obtained by contacting the corresponding author.
